# Experts’ recommendations for the management of adult patients with cardiogenic shock

**DOI:** 10.1186/s13613-015-0052-1

**Published:** 2015-07-01

**Authors:** Bruno Levy, Olivier Bastien, Karim Bendjelid, Alain Cariou, Tahar Chouihed, Alain Combes, Alexandre Mebazaa, Bruno Megarbane, Patrick Plaisance, Alexandre Ouattara, Christian Spaulding, Jean-Louis Teboul, Fabrice Vanhuyse, Thierry Boulain, Kaldoun Kuteifan

**Affiliations:** 1 CHU Nancy, Service de Réanimation Médicale Brabois; Pôle Cardiovasculaire et Réanimation Médicale, Hopital Brabois, 54511 Vandoeuvre-les-Nancy, France; 2 Service d’anesthésie cardiothoracique, CHU de Lyon, 28, boulevard Pinel, 69677 Bron, France; 3 Intensive Care Service, Geneva University Hospitals, Geneva Medical School, 1211 Geneva, Switzerland; 4 Medical Intensive Care Unit, Cochin Hospital, Assistance Publique-Hôpitaux de Paris 27 rue du Faubourg Saint Jacques, 75014 Paris, France; 5 SAMU-SMUR. CHU Nancy, Hôpital Central, 54000 Nancy, France; 6 Université Pierre et Marie Curie, Medical-Surgical Intensive Care Unit, iCAN, Institute of Cardiometabolism and Nutrition, Hôpital de la Pitié-Salpêtrière, Assistance Publique-Hôpitaux de Paris, Paris, France; 7 University Paris Diderot, Sorbonne Paris Cité; APHP, Lariboisière Saint Louis University Hospitals, Paris, France; 8 Réanimation Médicale et Toxicologique, Hôpital Lariboisière, INSERM U1144, Université Paris-Diderot, Paris, France; 9 Univ Diderot, Sorbonne Paris Cité, UMRS 942, AP-HP, Hôpital Lariboisière, Services des Urgences, F-75018 Paris, France; 10 CHU de Bordeaux, Service d’Anesthésie-Réanimation II Bordeaux, Univ. Bordeaux, Bordeaux, France; 11 Département de cardiologie, Hôpital européen Georges Pompidou, Assistance Publique Hôpitaux de Paris, Université Paris Descartes, Centre d’expertise de la mort subite, INSERM U 970, Paris, France; 12 Medical ICU, CHU de Bicêtre, Université Paris Sud, 78 rue du Général Leclerc, 94270 le Kremlin Bicêtre, France; 13 Service de Chirurgie Cardiaque et Transplantations, Hopital Brabois, CHU Nancy, Vandoeuvre-les-Nancy, France Université de Lorraine, Nancy, France; 14 CHR d’Orleans, Service de Réanimation Polyvalente, Hôpital de la Source, 14 avenue de l’hôpital, BP 6709 – 45067 Orléans, Cedex 02 France; 15 Service de Réanimation Médicale, Hôpital Emile Muller, 20, Avenue du Docteur René Laënnec, BP 1370-68100 Mulhouse, Cedex France

**Keywords:** Cardiogenic shock, Myocardial infarction, Monitoring, Extracorporeal membrane oxygenation

## Abstract

Unlike for septic shock, there are no specific international recommendations regarding the management of cardiogenic shock (CS) in critically ill patients. We present herein recommendations for the management of cardiogenic shock in adults, developed with the Grading of Recommendations Assessment, Development, and Evaluation (GRADE) system by an expert group of the French-Language Society of Intensive Care (Société de Réanimation de Langue Française (SRLF)), with the participation the French Society of Anesthesia and Intensive Care (SFAR), the French Cardiology Society (SFC), the French Emergency Medicine Society (SFMU), and the French Society of Thoracic and Cardiovascular Surgery (SFCTCV). The recommendations cover 15 fields of application such as: epidemiology, myocardial infarction, monitoring, vasoactive drugs, prehospital care, cardiac arrest, mechanical assistance, general treatments, cardiac surgery, poisoning, cardiogenic shock complicating end-stage cardiac failure, post-shock treatment, various etiologies, and medical care pathway. The experts highlight the fact that CS is a rare disease, the management of which requires a multidisciplinary technical platform as well as specialized and experienced medical teams. In particular, each expert center must be able to provide, at the same site, skills in a variety of disciplines, including medical and interventional cardiology, anesthesia, thoracic and vascular surgery, intensive care, cardiac assistance, radiology including for interventional vascular procedures, and a circulatory support mobile unit.

## Review

### Introduction

Contrary to septic shock, there are no international recommendations regarding the management of cardiogenic shock (CS) in intensive care. Reasons include the rarity of the disease, but also the fact that patients with CS often solely receive care in cardiology. Recent European Society of Cardiology (ESC) guidelines on the management of acute heart failure include a section on CS [1]. However, these guidelines are vague for the intensivist and at times obsolete (with regard to vasopressors, for example). The lack of a specific approach to the management of severe forms of CS in the latest international guidelines led our group to draw the following recommendations. The expert panel also deemed of interest to take into consideration the care spectrum for these specific patients, by emphasizing the notion of expertise.

### Methodology

These recommendations were drawn by an expert panel convened by the French Intensive Care Society (SRLF). The various disciplines contributing to the management of CS in adults were represented as follows: intensive care, anesthesia, cardiac surgery, cardiology, and emergency medicine. The organizing committee first defined, with the coordinator of the expert panel, the issues to be covered and then designated the experts in charge of each issue. The GRADE (Grading of Recommendations Assessment, Development and Evaluation) system and a literature analysis were used to formulate the recommendations [2]. A level of proof was defined for each bibliographic reference depending on the type of study. This level of proof could be reassessed taking into account the methodological quality of the study. The bibliographic references common to each assessment criterion were then combined. An overall level of proof was determined for each assessment criterion taking into account the levels of proof of each of the references, the consistency of the results between the different studies, the direct or indirect nature of the proof, the cost analysis, etc. A “strong” level of proof enabled formulation of a “strong” recommendation (should be performed, should not be performed, etc.). A “moderate”, “weak”, or “very weak” level of proof led to the formulation of an “optional” recommendation (should probably be performed, should probably not be performed, etc.). The proposed recommendations were presented and discussed one by one. The aim was not necessarily to reach a single and convergent opinion for all proposals, but rather to highlight points of agreement and points of disagreement or indecision. Each recommendation was then scored by each of the experts on a scale of one (complete disagreement) to nine (complete agreement). The collective scoring was established using a methodology derived from the RAND/UCLA appropriateness method; after elimination of the extreme values (outliers), the median of values attributed to each proposed recommendation defined agreement between the experts when the median value was between seven and nine, disagreement between one and three, and indecision between four and six. The agreement, disagreement, or indecision was “strong” if all values were within one of the following three ranges, (1–3), (4–6), or (7–9), and “weak” if the range of values straddled two ranges. In the absence of strong agreement, the recommendations were reformulated and again scored with the purpose of achieving a better consensus.

#### Cardiogenic shock definition

Cardiogenic shock (CS) is defined as a state of critical end-organ hypoperfusion due to reduced cardiac output. Notably, CS forms a spectrum ranging from mild hypoperfusion to profound shock.

Established criteria for the diagnosis of CS are as follows: (1) systolic blood pressure less than 90 mmHg for 30 min or mean arterial pressure less than 65 mmHg for 30 min or vasopressors required to achieve a blood pressure ≥90 mmHg; (2) pulmonary congestion or elevated left-ventricular filling pressures; (3) signs of impaired organ perfusion with at least one of the following criteria: (a) altered mental status; (b) cold, clammy skin; (c) oliguria; (d) increased serum lactate.

#### Area 1: epidemiology

1. In cardiogenic shock, a coronary cause should routinely be sought (strong agreement).

In over 70 % of cases, CS is related to acute myocardial infarction (MI) with ST-segment elevation, with or without mechanical complications (rupture of the septum, of the ventricular wall or of chordae tendineae). However, shock rarely occurs during MI (4.2 to 8.6 % of cases, depending on the study) and is declining [3]. ECG, troponin assay (repeated if necessary), and natriuretic peptide assay should be performed routinely, and coronary angiography should be considered. Patients in shock or with a high risk of CS should be hospitalized, preferably in intensive care. Except for patients with ventricular assistance, CS is associated with an immediate mortality of approximately 40 % [4]. Survivors have a very good prognosis, with a very good quality of life.

#### Area 2: cardiogenic shock in acute myocardial infarction

1. Predictors of progression to cardiogenic shock, in particular a heart rate >75 beats/min and signs of heart failure, should be sought in all patients with myocardial infarction (strong agreement).

In two-thirds of cases, shock is not present at admission and occurs in the first 48 h following admission for MI [5]. The predictors of ischemic CS are age, heart rate on admission above 75 beats/min, diabetes, history of MI, coronary artery-bypass surgery, the presence of signs of heart failure at admission, and anterior location of the infarction [6].

2- Coronary angiography, followed by coronary revascularization using angioplasty or exceptionally coronary artery-bypass surgery, is required in cardiogenic shock secondary to acute myocardial infarction irrespective of the time interval since the onset of pain (strong agreement).

The SHOCK trial conducted from April 1993 to November 1998 in 30 centers, and the associated registry assessed the effects of early revascularization (angioplasty or coronary artery-bypass surgery performed in the first 6 h) compared with stabilization by medical treatment in patients hospitalized for post-myocardial infarction CS. Whereas mortality at 30 days did not differ significantly in the early revascularization group compared with the control group (46.7 vs. 56 %) [7], early revascularization reduced mortality at 1 year (50.3 vs. 63.1 %, *p* = 0.027) [8] and 6 years [9]. The SHOCK trial showed no benefit of revascularization in the over-75 age group, although this subgroup study was limited by the small number of patients. (Certain, Some) registries suggest a benefit of revascularization in patients over 75 years of age.

Despite the fact that early revascularization is more efficient and given that CS secondary to MI has a poor prognosis, the ESC guidelines on MI recommend revascularization regardless of the time interval since the onset of the infarction. The ESC guidelines suggest the use of thrombolysis if angioplasty cannot be performed quickly (within 2 h), with secondary transfer to a center with a coronary angioplasty and cardiac surgery unit [10].

Intensive care before coronary angiography of CS secondary to MI should be of the “scoop and run” type. What is important is to transfer the patient alive to the coronary angiography unit without any delay as a result of an attempt at stabilization.

3- Cardiogenic shock secondary to acute myocardial infarction (MI) or MI likely to progress to cardiogenic shock should be managed in expert centers with a department of interventional cardiology and cardiac surgery fully equipped with cardiac support or in a coronary angioplasty center working in network with an expert center so as to plan a possible secondary transfer after emergency revascularization (strong agreement).

Patients presenting CS secondary to MI will generally receive multidisciplinary care. Coronary angioplasty is the reference treatment. Cardiac surgery is an integral part of the treatment of post-MI CS, through emergency bypass surgery, treatment of mechanical complications, or possible implantation of cardiac assistance devices. It is therefore desirable to transfer patients with post-MI CS or with MI plus predictors of secondary CS to an expert center, ideally one that offers interventional cardiology, cardiac surgery with the possibility of cardiac support, and even heart transplantation. If the distance is too great (>2 h between the first contact and artery clearing), patients with post-MI CS or with MI plus signs of CS should be transferred to an interventional cardiology department without cardiac surgery that works in network with an expert center, so as to plan any secondary transport needed after emergency angioplasty.

#### Area 3: monitoring in intensive care

1. An arterial catheter should be placed to monitor blood pressure (strong agreement).

The arterial catheter provides a continuous reading of diastolic arterial pressure (DAP), the driving pressure during relaxation and dilatation of the ventricles when filling with blood. DAP is influenced by peripheral arterial tone, heart rate, and arterial compliance. In a patient without bradycardia, low DAP is generally linked to a drop in arterial tone and calls for the use of a vasopressor or an increase in its dosage if mean arterial pressure is <65 mmHg.

2- Plasma lactate should be assayed repeatedly (in the absence of epinephrine therapy) to assess the persistence or correction of shock during treatment (strong agreement).
3- Organ function markers should be repeatedly checked (kidney, liver) (strong agreement).
4- A central venous catheter placed in the superior vena cava should be used for intermittent (blood sample) or continuous (fiber optic) measurement of central venous oxygen saturation (ScvO_2_) (strong agreement).

Inasmuch as central venous catheterization is mandatory in shock, particularly in cardiogenic shock, use of this catheter is recommended for intermittent (blood sample) or continuous (fiber optic) measurement of central venous oxygen saturation (ScvO_2_), which indicates whether cardiac output is appropriate to the metabolic conditions and provides useful information for adaptation of treatment.

5- Central venous pressure should not be measured because of the constraints of measurement and its limits as a marker of preload and of preload dependency (weak agreement).
6- In shock refractory to empirical treatment, cardiac output as well as mixed venous oxygen saturation (SvO_2_) or ScvO_2_ should be continuously monitored (strong agreement).
7- We suggest pulmonary artery catheterization in patients with refractory cardiogenic shock and right ventricular dysfunction (weak agreement).
8- We suggest the use of a transpulmonary thermodilution monitor/pulse wave analysis plus measurement (continuous or intermittent) of mixed venous oxygen saturation (SvO_2_) or ScvO_2_ when cardiogenic shock is refractory to initial treatment, in the absence of mechanical assistance and of predominant right ventricular dysfunction (weak agreement).
9- Routine echocardiography (transthoracic and/or transesophageal) should be used to identify the cause of (cardiogenic) shock, for later hemodynamic evaluations, and for the detection and treatment of complications (tamponade) (strong agreement).

#### Area 4: management of blood pressure and cardiac output in intensive care

1. A MAP of at least 65 mmHg should be reached using inotropic treatment and/or vasopressor treatment, or higher when there is a history of hypertension.

In analogy to septic shock, the target mean arterial pressure should be titrated to 65–70 mmHg, as a higher blood pressure is not associated with beneficial outcome with the exception of previously hypertensive patients.

2- Norepinephrine should be used to restore perfusion pressure during cardiogenic shock (strong agreement).

The only randomized study comparing two types of vasopressors—norepinephrine and epinephrine—showed that, for the same hemodynamic efficacy, epinephrine was associated with a higher heart rate, more arrhythmia, and lactic acidosis [11]. In a cohort study, De Backer et al. reported a reduction in mortality with norepinephrine when compared with dopamine [12]. Lastly, norepinephrine-induced increase in blood pressure in patients with post-MI CS is associated with an increase in cardiac index without an increase in heart rate and with increased SvO_2_ and reduced blood lactate [13].

3- Epinephrine can be a therapeutic alternative to the combination of dobutamine and norepinephrine, but is associated with a greater risk of arrhythmia, tachycardia, and hyperlactatemia (weak agreement).

In terms of hemodynamic effect, epinephrine clearly increases cardiac output, essentially by a heart rate effect, but is associated with severe hyperlactatemia of metabolic origin that hampers interpretation of lactate as a marker of the adequacy of tissue perfusion [14].

4- Dobutamine should be used to treat low cardiac output in cardiogenic shock (strong agreement).

In terms of hemodynamic and/or metabolic endpoints, compared with epinephrine, dobutamine results in less arrhythmia, less myocardial oxygen consumption, and a lower increase in lactate concentration [11]. Compared with dopamine, the increase in cardiac output is less marked with dobutamine, but at the cost of a greater drop in blood pressure [15]. Dobutamine must be used at the lowest possible dose, starting at 2 μg/kg/min. Its titration is based on cardiac index and SvO_2_. Dopamine must never be used.

5- Phosphodiesterase inhibitors or levosimendan should not be used first-line (strong agreement).

However, these drug classes, and in particular levosimendan, can improve the hemodynamics of patients with cardiogenic shock refractory to catecholamines. There is a pharmacological rationale for the use of this strategy in patients on chronic beta-blocker treatment (weak agreement).

CS refractory to catecholamines can be treated by perfusion of phosphodiesterase inhibitors [16] or levosimendan [17]. While these two drug classes improve macrocirculatory hemodynamics, only levosimendan appears to improve prognosis [17]. Levosimendan improves myocardial performance by increasing myofilament calcium sensitivity, without raising intracellular calcium and AMP concentrations. It therefore does not increase or has very little myocardial oxygen consumption. There is a pharmacodynamic rationale for the use of levosimendan in patients on chronic beta-blocker treatment. In CS refractory to catecholamines, it seems logical to consider the use of circulatory support rather than increased pharmacological support.

#### Area 6: prehospital and emergency care

1. In prehospital care of shock without an obvious cause, cardiogenic shock should be suspected, and 12-lead ECG should be performed (strong agreement).
2. In prehospital care, high diastolic blood pressure suggests a decrease in ventricular ejection evoking hypovolemia or heart failure (strong agreement).
3. In prehospital care, in the absence of signs of acute pulmonary edema or right ventricular overload, careful volume expansion is called for (strong agreement).
4. In prehospital and emergency care, the vasopressor of choice is norepinephrine (weak agreement).
5. There are no particular characteristics with regard to indications for intubation and assisted ventilation, except for right ventricular infarction (relative contraindication) (weak agreement).
6. Coronary angiography should be performed according to a defined protocol (call number, direct admission, ideally presence of an intensivist, post-procedure bed) (strong agreement).
7. Medical emergency call centers should transfer patients to an expert center (strong agreement).

#### Area 7: cardiogenic shock and cardiac arrest

1. Because of its high prevalence, a cardiogenic cause should routinely be sought using echocardiography during post-cardiac arrest care (strong agreement).

Early mortality is seen in a large proportion of patients admitted to intensive care after cardiopulmonary resuscitation because of shock [18]. This post-cardiac arrest shock comprises cardiogenic and vasoplegic components, although myocardial failure is sometimes overlooked, particularly when the cause of cardiac arrest is non cardiac in origin. This myocardial failure occurs early, is severe, generally resolves within 48 h, and is secondarily associated with a strong vasoplegic component, the result of inflammation syndrome, which is well documented in post-cardiac arrest [19]. Microcirculatory anomalies are associated with this clinical picture [20].

2- In proven post-cardiac arrest cardiogenic shock, especially in shockable rhythms, routine coronary angiography is recommended (strong agreement).

Ischemic cardiopathy, in a setting of acute coronary syndrome or chronic cardiopathy, is currently the leading reason for cardiopulmonary resuscitation in the Western world. Bearing this in mind, several authors have proposed routine coronary angiography in the management of cardiac arrest of presumed cardiac cause, so as to detect, and if need be treat, acute coronary occlusion. Several studies have reported that coronary angioplasty is performed in between 26 and 50 % of cases at the initial phase of a cardiac arrest (on the principle that angioplasty is performed when acute coronary occlusion is deemed responsible for the cardiac arrest). Contrasting with these high rates of coronary occlusion, noninvasive strategies for detection of acute coronary occlusion are, at present, disappointing. ST-segment changes and troponin increase are insufficiently discriminatory compared with the potential benefit of properly conducted coronary revascularization.

3- The existence or onset of post-cardiac arrest cardiogenic shock is not a contraindication to therapeutic hypothermia (strong agreement).
4- During post-cardiac arrest cardiogenic shock, hyperthermia *must be avoided* (strong agreement).

#### Area 8: circulatory support

1. Intraaortic balloon counterpulsation should not be used in cardiogenic shock in the setting of myocardial infarction managed effectively and quickly by angioplasty (weak agreement).

In the randomized multicenter IABP-SHOCK II trial [4, 21], there was no significant difference in mortality at day 30 (39.7 % in the intraaortic balloon counterpulsation group and 41.3 % in the control group, relative risk 0.96, 95 % confidence interval 0.79–1.17, *p* = 0.69) in the total population or in any of the subgroups, in particular patients with systolic blood pressure <80 mmHg. The final 12-month results of this cohort were recently reported [22] as follows: 155 (52 %) of the 299 patients in the intraaortic balloon counterpulsation group and 152 (51 %) of the 296 control patients had died (relative risk 1.01, 95 % confidence interval 0.86-1.18, *p* = 0.91). Complications were identical in the two groups. According to the ESC recommendation, intraaortic balloon counterpulsation can be used in the case of revascularization by thrombolysis or in the absence of initial revascularization or if rescue therapy such as extracorporeal membrane oxygenation, the Impella® device, or the TandemHeart™ percutaneous ventricular assist device is not available on site.

2- If temporary circulatory support is needed, the use of peripheral extracorporeal membrane oxygenation is preferred (strong agreement).

The veno-arterial extracorporeal membrane oxygenation (ECMO) circuit, which comprises a centrifugal pump and a membrane oxygenator, provides complete cardiopulmonary support. ECMO reduces ventricular preload, but increases ventricular afterload. ECMO is currently the cheapest and longest-lasting device and the only device to enable complete respiratory support in addition to circulatory support. It is easier to set up than TandemHeart™ or Impella® 5.0. ECMO can be rapidly implemented at bedside, even in the field, when a mobile ECMO unit is used. Numerous studies have reported the use of ECMO in refractory CS in the case of MI [22, 23] or myocarditis [23–25], after cardiac surgery [26, 27] and in the case of refractory cardiac arrest [28, 29]. There is no meta-analysis or randomized trial that has assessed prognosis after ECMO in CS. A single retrospective study of before/after design [30] has shown an improved prognosis after implementation of ECMO in refractory CS in MI (mortality; 18/25, 72 % before ECMO and 18/46, 39 % after, *p* = 0.003).

3- The Impella® 5.0 device can be used in the management of cardiogenic shock complicating myocardial infarction if the surgical team has experience with its placement (weak agreement).

On the contrary, due to the limited blood blow obtained with the Impella® 2.5 device, the latter is not recommended for cardiac support during cardiogenic shock.

4- The creation of circulatory support mobile units to set up veno-arterial ECMO in the field before transfer of the patient to an expert center is recommended (strong agreement).

When a CS patient’s clinical condition is deemed too precarious to allow transfer without circulatory support, a mobile unit should be used to set up ECMO quickly in the department where the patient is treated before transfer of the patient to the expert center. There is no meta-analysis or randomized trial to date assessing this strategy. Some retrospective studies have reported that this strategy improved patient prognosis, demonstrating its feasibility and equivalence of prognosis compared with patients managed in hospital.

#### Area 9: general treatments

1. In cardiogenic shock, ventricular resynchronization is possible in cases of left-bundle branch block with wide QRS complex (weak agreement).

ESC recommendations do not mention CS as an indication for resynchronization. Resynchronization has enabled improvement of clinical condition and weaning from inotropic agents in patients with severe acute heart failure (NYHA class III-IV). In 15 CS patients with left-bundle branch block, a German team showed that temporary resynchronization of the left ventricle using a lead placed in the coronary sinus (while maintaining atrioventricular synchronous contraction using a right atrial lead) optimized macrocirculatory parameters and reduced blood-lactate concentration [31]. However, because of the absence of literature data of high level of proof, its routine use in CS patients cannot be recommended, because of the risk of infectious and mechanical complications associated with pacemaker implantation.

2- In patients with cardiogenic shock and arrhythmia (atrial fibrillation), restoration of sinus rhythm, or slowing the heart rate if restoration fails, can prove useful (strong agreement).
3- In cardiogenic shock, antithrombotic drugs at the usual dosages should be used, in their recognized indications, bearing in mind that the hemorrhagic risk is greater in this situation (strong agreement).

The only exception is that antiplatelet agents such as clopidogrel or ticagrelor should be given after elimination of a surgical complication, i.e., not in a prehospital setting.

4- Nitrovasodilators should not be continued or introduced during cardiogenic shock (strong agreement).
5- When cardiogenic shock is associated with pulmonary edema, diuretics can be continued or introduced (weak agreement).
6- Beta-blockers should not be used during cardiogenic shock (strong agreement).
7- In ischemic cardiogenic shock, the hemoglobin level should be maintained at around 10 g/dL during the acute phase (weak agreement).

During the very acute phase of cardiogenic shock in which oxygen delivery is low and cardiac output is insufficient to meet metabolic demand, it may be useful to increase hemoglobin levels. Nevertheless, as soon as cardiogenic shock is resolved, the transfusion trigger should to 7 g/dL.

8- When cardiogenic shock is not ischemic in origin, the hemoglobin level should be maintained above 8 g/dL (weak agreement).

#### Area 10: surgery and cardiogenic shock

1. In adults presenting severe aortic stenosis associated with cardiogenic shock, the aortic stenosis should be managed, probably by valvuloplasty and if need be, under ECMO (strong agreement).

In a CS patient presenting with severe aortic stenosis, and according to international recommendations [32], treatment of the aortic obstacle should be envisaged. The surgical treatment of severe aortic stenosis complicated by CS carries a high surgical risk. Literature data evaluating surgical results in this indication are scarce [33]. Surgeons are often disinclined to operate on these hemodynamically unstable patients. To reduce this surgical risk, aortic valvuloplasty-“pending” surgery can be envisaged. Literature studies only relate to retrospective cohorts with a limited number of patients in which the results of aortic valvuloplasty in CS patients are evaluated. In these studies, hospital mortality following aortic valvuloplasty in CS patients range between 42 and 57 %. Serious complications occur in 10 % of cases, and valvular stenosis associated with clinical deterioration recurs in 6 to 12 months.

2- In adults presenting with severe aortic stenosis responsible for cardiogenic shock, valve repair/replacement should not be performed by first-line transcatheter aortic valve implantation (weak agreement).

Percutaneous valve placement is currently contraindicated in CS patients, although a retrospective study with encouraging results has been published (hospital mortality 19 %) [34].

3- In adults presenting with aortic or mitral insufficiency responsible for cardiogenic shock, the valve should be replaced without delay (strong agreement).
4- In adults presenting with mitral insufficiency responsible for cardiogenic shock, intraaortic balloon counterpulsation and vasoactive/cardiac drugs can be used to achieve stabilization pending surgery, which should be performed promptly (<12 h) (strong agreement).
5- In the case of interventricular communication, the patient should be transferred to an expert center for assistance and to discuss surgery (strong agreement).
6- It is possible to use milrinone or levosimendan as an alternative to dobutamine in second-line treatment of cardiogenic shock after cardiac surgery (weak agreement). Levosimandan could be used as a first-line treatment of cardiogenic shock after coronary bypass surgery (weak agreement).

Because they do not act on beta-receptors, these two drugs are potentially useful in cardiogenic management. However, because of their prolonged and potential vasodilatatory actions, these two drugs should be handled with care. Levosimendan is the only drug for which a randomized study indicates a significant reduction in mortality in treatment of CS after coronary artery-bypass surgery, in comparison with dobutamine. A meta-analysis on levosimendan and cardiac surgery also indicated a significant reduction in mortality, although the analysis indiscriminately included studies in which levosimendan was administered prophylactically or before or during surgery and studies in which administration of levosimendan was initiated after surgery in response to CS [35]. Other studies of very low level of proof did not find any reduction in mortality with levosimendan after cardiac surgery (mixed cohort of patients with coronary artery-bypass surgery and valve replacement). It therefore appears possible to administer levosimendan to treat CS after coronary artery-bypass surgery.

7- It is possible to use milrinone as first-line treatment to increase inotropism in cardiogenic shock associated with right ventricular failure (weak agreement).
8- It is possible to administer levosimendan after coronary artery-bypass surgery as first-line treatment of cardiogenic shock after cardiac surgery (weak agreement).

#### Area 11: management of cardiac drug toxicity leading to cardiogenic shock

1. Knowledge of the causal mechanism(s) (hypovolemia, vasodilation, altered contractility) is essential for adaptation of treatment. Emergency echocardiography is mandatory, followed by continuous measurement of cardiac output and SvO_2_ (strong agreement).

Broadly speaking, a distinction should be drawn between toxicity with hypokinesis in cardiogenic shock and vasoplegic shock. The latter is generally treatable using vasopressor drugs (norepinephrine) and volume expansion. The possibility of mixed forms or of vasoplegic forms progressing to hypokinesis should not be overlooked.

2- When there is cardiac drug toxicity in a state of shock, emergency echocardiography is required to detect a hypokinetic state (strong agreement).
3- Cardiac toxicity (particularly associated with sodium channel blockers, calcium inhibitors, and beta-blockers) in a state of shock should prompt transfer of the patient to an expert center with experience in ECMO, especially if echocardiography reveals a hypokinetic state. In shock that is refractory or progresses rapidly and occurring in a center without ECMO, use of a circulatory support mobile unit should be considered. Ideally, ECMO should be implemented before onset of other organ failures (liver, kidney, ARDS) and in all cases before cardiac arrest. Vasoplegic shock alone is not an indication for ECMO (strong agreement).
4- Heart failure necessitates the addition of dobutamine to norepinephrine or the use of epinephrine, bearing in mind its side effects (lactic acidosis) (strong agreement).
5- Adjuvant treatments such as glucagon (beta-blockers), insulin therapy (calcium inhibitors), and lipid emulsion (lipid-soluble cardiotoxic local anesthetic) should be initially used in association with vasopressor/inotrope agents (strong agreement). Medical supportive treatment should not delay use of ECMO in case of refractory shock (weak agreement).
6- The administration of molar sodium bicarbonate (doses fractionated from 100 to 250 mL up to a maximum total dose of 750 mL) is indicated in toxic shock with intraventricular conduction abnormalities (wide QRS complex), together with other treatments (strong agreement).

#### Area 12: cardiogenic shock complicating end-stage heart disease

1. Patients with severe chronic heart disease should be assessed for their heart transplant eligibility in a center equipped for this intervention (strong agreement).
2. A patient with end-stage decompensated heart failure considered eligible for heart transplantation should be rapidly managed in the expert center that conducted the assessment (strong agreement).
3. ECMO/extracorporeal life support is indicated as first-line treatment in the case of progressive or refractory shock (persistent lactic acidosis, low cardiac output, high doses of catecholamines, kidney and/or liver failure) and of cardiac arrest without “no flow” in patients with advanced chronic heart disease with no contraindication for heart transplantation (strong agreement).
4. In progressive or refractory cardiogenic shock in a patient hospitalized for decompensated heart failure in a center without circulatory support, prompt use of a circulatory support mobile unit to implement veno-arterial ECMO followed by transfer of the patient on ECMO to an expert center is recommended (strong agreement).

#### Area 13: post-shock treatment

1. Once the acute phase of cardiogenic shock has been managed, appropriate oral treatment of heart failure should be implemented and closely monitored (strong agreement).
2. Very early after withdrawal of vasopressor drugs, treatment with beta-blockers, angiotensin-converting enzyme inhibitors, and aldosterone antagonists should be introduced to improve survival by reducing the risk of arrhythmia and of recurrence of an episode of heart failure (strong agreement).

Once shock is treated, management of the patient should follow the most recent guidelines for treatment of chronic heart failure. Treatment is to be introduced early, upon withdrawal of vasopressor drugs at small doses that are progressively raised. In certain cases, introduction of treatment is poorly tolerated, and temporary vasopressor reintroduction may be necessary.

#### Area 14: other etiologies

1. Patients with acute heart failure or cardiogenic shock associated with myocarditis should be transferred to an expert center, on ECMO if necessary (strong agreement).
2. Bromocriptine treatment should be considered in cardiogenic shock complicating peripartum cardiomyopathy after discounting pre-existing or genetic heart disease (weak agreement).
3. Before making a diagnosis of stress cardiomyopathy, coronary artery disease should be discounted by an imaging technique (coronary angiography or computed tomography) and ventricular imaging (echocardiography or ventriculography or magnetic resonance imaging) (strong agreement).
4. Treatment of severe stress cardiomyopathy is based on management of any predisposing factor and should be symptomatic, based on restoring a favorable myocardial energy balance (strong agreement).
5. In stress cardiomyopathy, a decrease in dosage of inotropic agents or even their discontinuation is recommended, provided circulatory flow rate can be controlled by mechanical means in severe shock such as: intraaortic balloon counterpulsation and short-term circulatory support (ECMO, TandemHeart™, centrifugal pump). As prognosis is good and reversibility is often quick, the risk-benefit ratio must be evaluated by an expert center (strong agreement).

#### Area 15: medical care pathway

This is probably the most important aspect of our expert panel’s recommendations. CS is a rare disease for which management requires a multidisciplinary technical platform and specialized and experienced medical teams.

1. From emergency to rehabilitation, the patient must be managed along a specialized medical care pathway suited to the actual or potential seriousness of his or her condition (strong agreement).
2. This medical care pathway must be fully recognized by all those involved (emergency medical services, emergency room, cardiology, intensive care, cardiac surgery departments). In particular, an expert-center 24/7 telephone line offering specialist advice must be available (strong agreement).
3. Official recognition by the regional health authorities of expert centers is recommended. Each expert center must be able to provide, at the same site, skills in a variety of disciplines (medical and interventional cardiology, anesthesia, thoracic and vascular surgery, intensive care, radiology including for interventional vascular procedures, circulatory support mobile unit) (strong agreement).

## Abbreviations

BP: blood pressure
CS: cardiogenic shock
ECMO: extracorporeal membrane oxygenation
MI: myocardial infarction
ScvO_2_: central venous oxygen saturation
