# Erratum to: Experts’ recommendations for the management of adult patients with cardiogenic shock

**DOI:** 10.1186/s13613-015-0063-y

**Published:** 2015-09-22

**Authors:** Bruno Levy, Olivier Bastien, Karim Bendjelid, Alain Cariou, Tahar Chouihed, Alain Combes, Alexandre Mebazaa, Bruno Megarbane, Patrick Plaisance, Alexandre Ouattara, Christian Spaulding, Jean-Louis Teboul, Fabrice Vanhuyse, Thierry Boulain, Kaldoun Kuteifan

**Affiliations:** CHU Nancy, Service de Réanimation Médicale Brabois; Pôle Cardiovasculaire et Réanimation Médicale, Hopital Brabois, 54511 Vandoeuvre-les-Nancy, France; Service d’anesthésie cardiothoracique, CHU de Lyon, 28, boulevard Pinel, 69677 Bron, France; Intensive Care Service, Geneva University Hospitals, Geneva Medical School, 1211 Geneva, Switzerland; Medical Intensive Care Unit, Cochin Hospital, Assistance Publique-Hôpitaux de Paris, 27 rue du Faubourg Saint Jacques, 75014 Paris, France; SAMU-SMUR. CHU Nancy, Hôpital Central, 54000 Nancy, France; Université Pierre et Marie Curie, Medical-Surgical Intensive Care Unit, iCAN, Institute of Cardiometabolism and Nutrition, Hôpital de la Pitié-Salpêtrière, Assistance Publique-Hôpitaux de Paris, Paris, France; University Paris Diderot, Sorbonne Paris Cité; APHP, Lariboisière Saint Louis University Hospitals, Paris, France; Réanimation Médicale et Toxicologique, Hôpital Lariboisière, INSERM U1144, Université Paris-Diderot, Paris, France; Univ Diderot, Sorbonne Paris Cité, UMRS 942, AP-HP, Hôpital Lariboisière, Services des Urgences, 75018 Paris, France; CHU de Bordeaux, Service d’Anesthésie-Réanimation II Bordeaux, Univ. Bordeaux, Bordeaux, France; Département de cardiologie, hôpital européen Georges Pompidou, Assistance Publique Hôpitaux de Paris, Université Paris Descartes, centre d’expertise de la mort subite, INSERM U 970, Paris, France; Medical ICU, CHU de Bicêtre, Université Paris Sud, 78 rue du Général Leclerc, 94270 le Kremlin Bicêtre, France; Service de Chirurgie Cardiaque et Transplantations, Hopital Brabois, CHU Nancy, Vandoeuvre-les-Nancy, France Université de Lorraine, Nancy, France; CHR d’Orleans, Service de Réanimation Polyvalente, Hôpital de la Source, 14 avenue de l’hôpital, BP 6709-45067, Orléans Cedex 02, France; Service de Réanimation Médicale, Hôpital Emile Muller, 20, Avenue du Docteur René Laënnec, BP 1370-68100, Mulhouse Cedex, France

## Erratum to: Annals of Intensive Care (2015) 5:17 DOI 10.1186/s13613-015-0052-1

The originally published article [1] unfortunately contained several mistakes, these are detailed below. The author’s list was presented incorrectly in both the HTML and PDF versions of this article.

Bendjelid Karim’s name was incorrectly presented as Karim Benjelid.

Christian Spaulding’s name was incorrectly spelt as Christian Splaulding.

Christian Spaulding’s affiliation was incorrectly presented as “Inserm U 970, Département de cardiologie, centre d’expertise de la mort subite, Université Paris-Descartes, hôpital européen Georges-Pompidou, AP-HP, Paris, France”, the correct affiliation is “Département de cardiologie, hôpital européen Georges Pompidou, Assistance Publique Hôpitaux de Paris, Université Paris Descartes, centre d’expertise de la mort subite, INSERM U 970, Paris”.

The original article has been updated to reflect this change.
